# Comparative mitochondrial genome brings insights to slight variation in gene proportion and large intergenic spacer and phylogenetic relationship of mudskipper species

**DOI:** 10.1038/s41598-024-52979-4

**Published:** 2024-02-09

**Authors:** Valdemiro Muhala, Aurycéia Guimarães-Costa, Adam Rick Bessa-Silva, Luan Pinto Rabelo, Jeferson Carneiro, Isadola Eusébio Macate, Luciana Watanabe, Oscar David Balcázar, Grazielle Evangelista Gomes, Marcelo Vallinoto, Iracilda Sampaio

**Affiliations:** 1 Laboratório de Evolução Bragança, Instituto de Estudos Costeiros, Universidade Federal do Pará, Pará, Brazil; 2 Divisão de Agricultura, Instituto Superior Politécnico de Gaza, Chokwe, 1204 Mozambique; 3 Laboratório de Genética Aplicada, Instituto de Estudos Costeiros, Universidade Federal do Pará, Bragança, Pará, Brazil; 4 Laboratório Associado, Campus agrário de Vairão, Centro de Investigação em Biodiversidade e Recursos Genéticos, Universidade do Porto, Vairão, Portugal

**Keywords:** Evolution, Genetics, Molecular biology, Zoology

## Abstract

Fish mitochondrial genome have been largely studied worldwide for evolutionary and other genetic purposes and the structure and gene organization are commonly conservative. However, several studies have demonstrated that this scenario may present variations in some taxa, showing differentiation on the gene rearrangement. In this study, the complete mitogenome of terrestrial fish *Boleophthalmus dussumier*i was generated and compared with other species of the Exudercidae fishes. The newly complete mitogenome generated is circular and 16,685 bp of length, and it contained 13 protein-coding genes (PCGs), two ribosomal RNA (rRNAs), 22 transfer RNA genes (tRNAs), and one control region (CR), with high conservative structure, like other Mudskippers. Most of the PCG showed similar codon usage bias. The gene length was found to be different specially for the CR, *12S rRNA* gene and *ND5* gene in some taxon. All the *Boleophthalmus* species showed a gene duplication in the CR, except for *B. dussumieri*, and they presented a long intergenic spacer specially on the *tRNA-Pro*/ OH Tandem duplication/random loss (TDRL) and dimer-mitogenome and nonrandom loss (DMNL) are suitable to explain the mitogenome rearrangement observed in this study. The phylogenetic analysis well supported the monophyly of all mudskipper species and the analysis positioned the *Periophthalmus* clade as the most basal of the terrestrial fishes. This finding provides basis and brings insights for gene variation, gene rearrangements and replications showing evidence for variety of mitochondrial structure diversity within mudskippers.

## Introduction

Mudskippers are amphibious fishes with peculiar characteristics, living in muddy areas of mangroves^1,2^. The genus *Boleophthalmus* is known to be one of the most terrestrial among mudskippers, exhibiting locomotory, respiratory, vertebral adaptation and specializations that enable overland excursions lasting up to 14 h^3,4^. Despite being a crucial group of fish for evolutionary and ecological studies, only seventeen species are available in literature (Table 2), three of which belong to the genus *Boleophthalmus*^5–8^.

Mitogenome studies enable a wide range of investigations, including tandem repeats, phylogenetic analyses, gene rearrangements, gene overlap, analysis short or long intergenic regions^9,10^, also including loss of genes^11–15^. The configuration, structure, and organization of the mitochondrial genome in many bony fishes exhibit a similar structural conformity with 37 genes, including 13 protein-coding genes (PCGs), 22 transfer RNAs (tRNAs) and 2 ribosomal RNA (rRNAs) genes, and one control region^16,17^. However, some organisms present variation in the length of genes (resulting in long gene sequences) and sometimes repetitions may be found in portions of the genes, including in the protein-coding genes^18^, or in the control region^19^.

Several studies have used tools to conduct comparative analysis, aiming to understand the correlations in terms of GC contents percentages, presence of complete or short gene repetition, protein-coding and non-coding regions, as well as the consistency between different regions^19,20^, which provides relevant information on proportions of changes in gene rearrangements within each class or family. These studies have provided valuable insights into the proportions of gene rearrangements within each class or family^11,21^.

Variations in mitogenome length and genetic organization are often associated with various evolutionary events, such as nucleotide insertions and deletions in the control region^22,23^, as well as duplications followed by deletion in certain regions, particularly on tRNAs^24^. This features directly impact functional differences, considering that the distinct gene structure influences gene function^25^.

The analysis of complete mitogenomes offers significant advantages in studying vertebrates, and researchers can utilize the available data in public databases, such as the NCBI, to address phylogenetic questions^11,21^.

Mudskippers exhibit variations in individual characteristics, encompassing both genetic variations and environmental adaptations^26–28^. Therefore, through research utilizing these tools, various mechanisms and genetic information can still be uncovered. Studies already carried out with mudskipper mitogenomes generally describe their complete mitogenomes, except for some studies that, in addition to studying phylogenetic relationships, they bring insights on the genetic functioning of the group^6–8,27,29,30^.

In this study, we present for the first time, the complete mitochondrial genome of the *B. dussumieri* (Valenciennes 1837), a mudskipper species. The primary objective of the study was first to describe the complete mitochondrial genome and present the comparative analysis with available mudskipper mitogenomes. Additionally, we aimed to reconstruct the phylogenetic relationships among the seventeen mitogenomes of individuals within the family, based on the thirteen protein-coding genes. Furthermore, we implemented a repeat sequence analysis in the control region to assess the potential presence of gene repetitions as well as duplication in CR and certain gene/regions.

## Results and discussions

### Mitogenome organization, composition, and skewness

**Figure 1:**
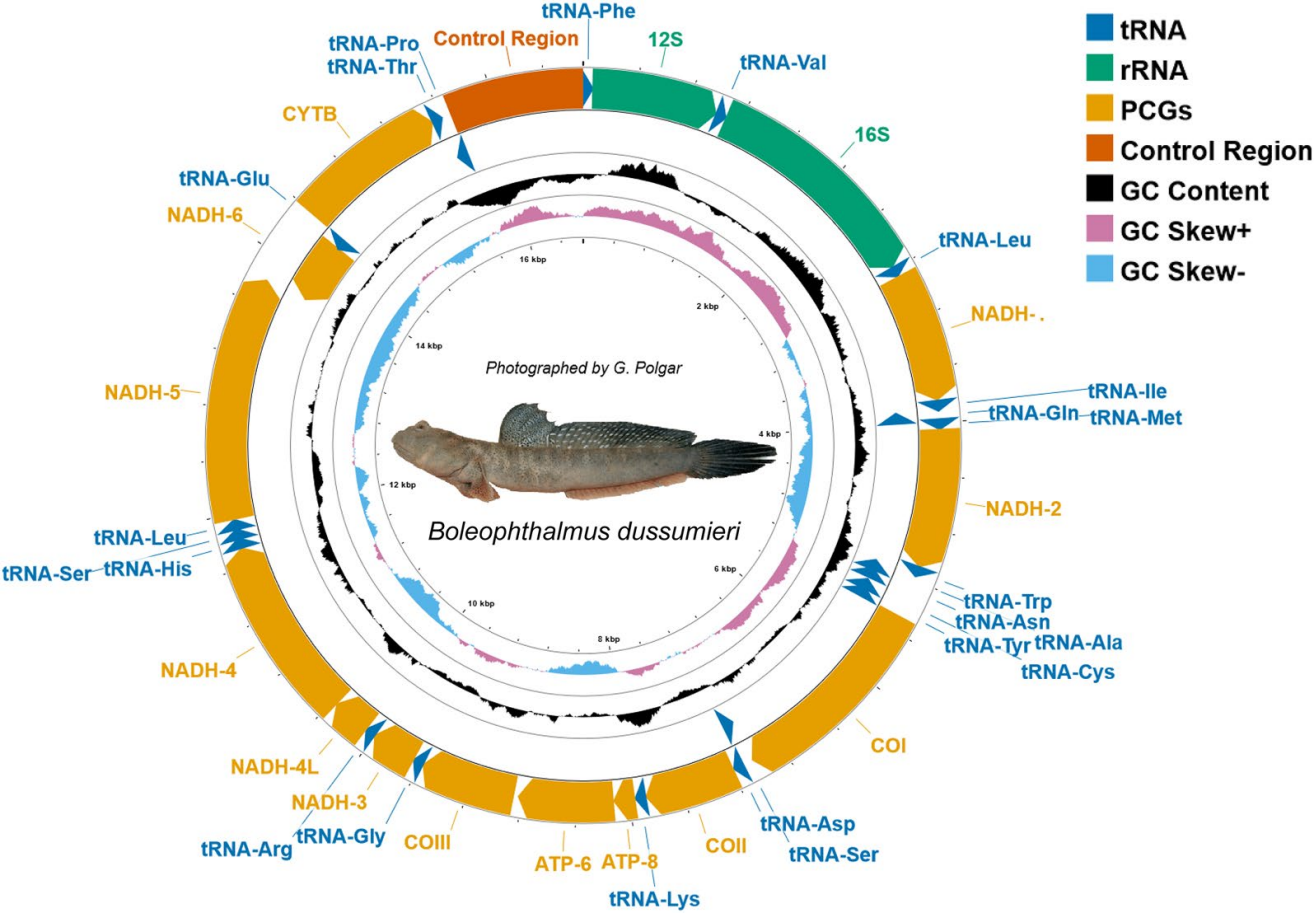
The mitochondrial genome map of *B. dussumieri* is represented by different colors, adapted for colorblind individuals following Wong’s protocol (2011). Gene groups are visually distinguished by their respective colors. The credit for the fish image goes to G. Polgar.

**Figure 2:**
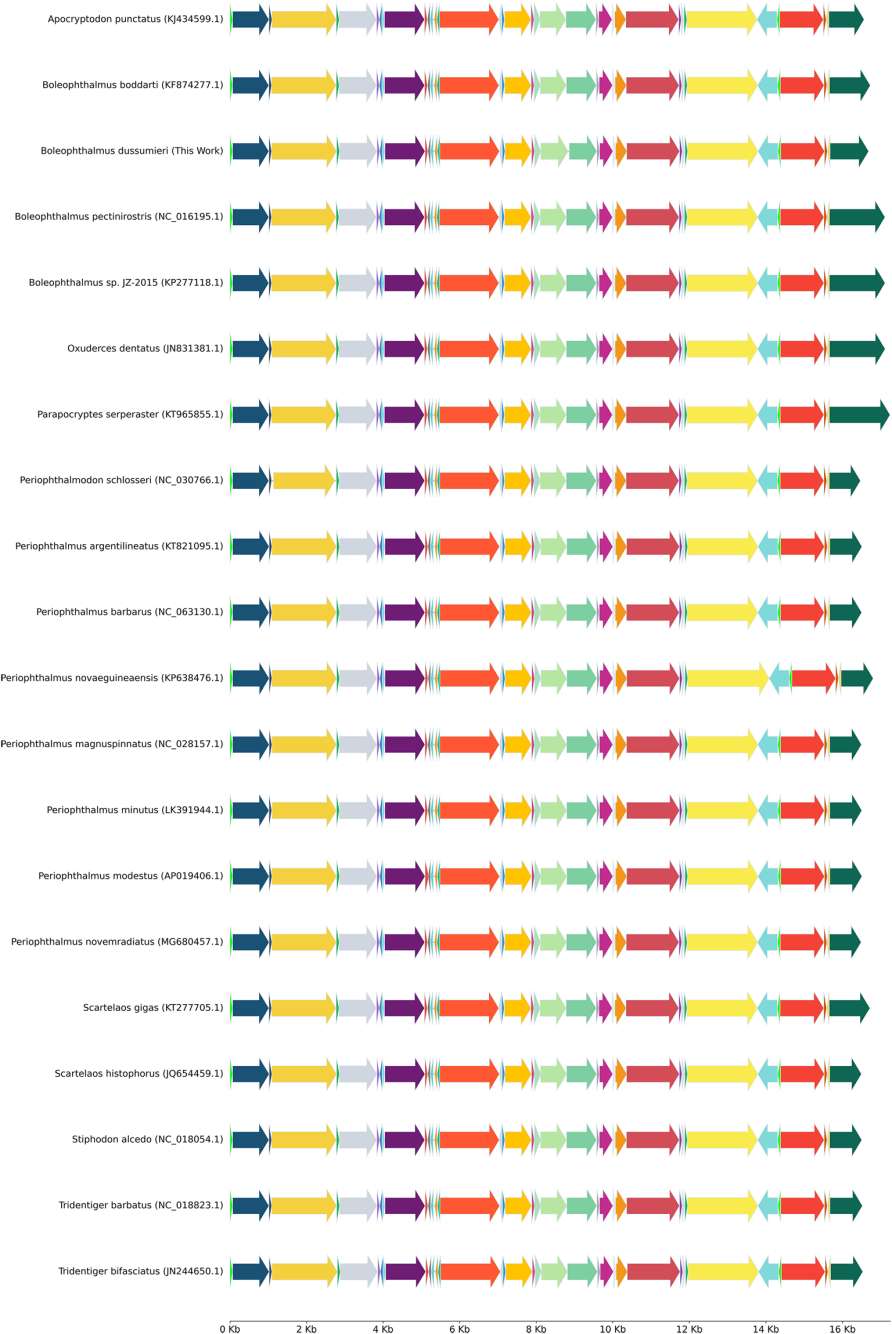
Gene order of the seventeen mudskippers and three goby fishes.

The complete mitochondrial genome of *B. dussumieri* is (GenBank accession **XX)** 16,685 bp of length, which is similar to other mudskippers mitogenomes (16,470–17,243 bp) (Tables 1 and 2). The mitochondrial genome and the structure were also typical of the mudskipper species and with highly conservative sites, comprising 37 mitochondrial genes (13 PCGs, 22 tRNAs, and 2 rRNAs) and one control region (CR) (Figs. 1 and 2). The mitogenome composition and features are presented in Tables 1 and 2, highlighting the characteristics of the mitogenome, including gene content and specific regions.

**Table 1:** Description of the mitogenome.

Gene	Position		Length (bp)	Intergenic region (bp)	Start codon/Stop codons	Strand
	From	To				
tRNA-Phe	1	68	68			H
12S	69	1016	948	0		H
tRNA-Val(tac)	1017	1088	72	51		H
16S	1140	2779	1640	8		H
tRNA-Leu2(taa)	2788	2862	75	0		H
ND1	2863	3837	975	4	ATG/TAA	H
tRNA-Ile (gat)	3842	3911	70	− 1		H
tRNA-Gln (ttg)	3911	3981	71	− 1		L
tRNA-Met(cat)	3981	4049	69	0		H
ND2	4050	5096	1047	0	ATG/TAA	H
tRNA-Trp (tca)	5097	5167	71	2		H
tRNA-Ala (tgc)	5170	5238	69	1		L
tRNA-Asn (gtt)	5240	5312	73	4		L
OL	5317	5347	31	0		H
tRNA-Cys(gca)	5348	5412	65	0		L
tRNA-Tyr(gta)	5413	5483	71	1		L
COI	5485	7038	1554	0	GTG/TAA	H
tRNA-Ser2(tga)	7039	7109	71	3		L
tRNA-Asp (gtc)	7113	7184	72	4		H
COII	7189	7879	691	0	ATG/T(AA)	H
tRNA-Lys(ttt)	7880	7955	76	1		H
ATP8	7957	8121	165	− 7	ATG/TAG	H
ATP6	8115	8800	686	117	ATG/TA(A)	H
COIII	8918	9570	653	14	ATG/TA(A)	H
tRNA-Gly (tcc)	9585	9656	72	0		H
NAD3	9657	10007	351	− 2	ATG/TAG	H
tRNA-Arg (tcg)	10006	10074	69	0		H
NAD4L	10075	10371	297	− 7	ATG/TAA	H
NAD4L	10365	11745	1381	0	ATG/T(AA)	H
tRNA-His (gtg)	11746	11814	69	0		H
tRNA-Ser 1(gct)	11815	11882	68	3		H
tRNA-Leu1(tag)	11886	11958	73	0		H
NAD5	11959	13797	1839	− 4	ATG/TAA	H
NAD6	13794	14318	525	− 3	ATG/TAG	L
tRNA-Glu (ttc)	14316	14384	69	5		L
CYTB	14390	15530	1141	0	ATG/T(AA)	H
tRNA-Thr (tgt)	15531	15603	73	1		H
tRNA-Pro (tgg)	15605	15674	70	495		L
OH	16170	16500	331	185		H
D-loop	15675	16684

**Table 2:** Composition and skewness.

Species	Whole genome						PCGs					rRNA					tRNA					Control region				
	Voucher	Size	GC%	AT%	GC-Skew	AT- Skew	Size	GC%	AT%	GC-Skew	AT- Skew	Size	GC%	AT%	GC-Skew	AT- Skew	Size	GC%	**AT%**	GC-Skew	AT- Skew	Size	GC%	AT%	GC-Skew	AT- Skew
*A. punctatus*	KJ434599.1	16,566	42.79	57.21	− 0.211	0.001	11,414	43.08	56.92	− 0.227	− 0.102	2630	43.65	56.35	− 0.075	0.221	1562	44.75	55.25	0.03	0.027	923	32.61	67.39	− 0.163	0.013
*B. boddarti*	KF874277.1	16,727	45.12	54.88	− 0.29	0.055	11,427	45.9	54.1	− 0.304	− 0.049	2636	46.17	53.83	− 0.129	0.26	1561	43.63	56.37	0.057	0.03	1067	35.8	64.2	− 0.262	0.104
*B. dussumieri*	This study	16,685	43.43	56.54	− 0.285	0.05	11,399	43.64	56.31	− 0.311	− 0.053	2647	45.11	54.89	− 0.111	0.251	1556	43.89	56.11	0.042	0.029	1010	34.65	65.35	− 0.194	0.088
*B. pectinirostris*	NC_016195.1	17,111	43.78	56.22	− 0.294	0.055	11,429	44.3	55.7	− 0.31	− 0.05	2633	45.5	54.5	− 0.12	0.254	1561	43.75	56.25	0.054	0.023	1453	36.06	63.94	− 0.324	0.104
*Boleophthalmus. *sp. *JZ− 2015*	KP277118.1	17,113	44.26	55.74	− 0.3	0.065	11,427	44.62	55.38	− 0.322	− 0.045	2633	46.18	53.82	− 0.128	0.26	1561	44.33	55.67	0.04	0.036	1456	37.29	62.71	− 0.285	0.161
*O. dentatus*	JN831381	17,116	41.95	58.05	− 0.279	0.039	11,428	42.44	57.56	− 0.297	− 0.061	2624	44.05	55.95	− 0.102	0.223	1565	44.41	55.59	0.05	0.018	1456	30.84	69.16	− 0.318	0.1
*P. argentilineatus*	KT821095.1	16,509	43.84	56.16	− 0.27	0.007	11,427	44.0	56.0	− 0.285	− 0.095	2646	45.12	54.88	− 0.111	0.231	1556	44.28	55.72	0.057	0.015	840	35.36	64.64	− 0.205	0.006
*P. barbarus*	NC_063130.1	16,502	43.16	56.84	− 0.263	0.014	11,427	43.48	56.52	− 0.275	− 0.083	2643	44.76	55.24	− 0.096	0.204	1555	43.6	56.4	0.053	0.031	839	32.3	67.7	− 0.255	0.063
*P. magnuspinnatus*	NC_028157.1	16,497	44.2	55.8	− 0.269	0.015	11,428	44.4	55.6	− 0.29	− 0.08	2644	44.82	55.18	− 0.105	0.225	1561	44.2	55.8	0.046	0.04	831	37.79	62.21	− 0.166	− 0.021
*P. minutus*	LK391944.1	16,506	44.8	55.2	− 0.269	0.01	11,442	45.24	54.76	− 0.282	− 0.093	2635	45.92	54.08	− 0.104	0.22	1554	44.21	55.79	0.057	0.022	839	34.68	65.32	− 0.196	− 0.011
*P. modestus*	AP019406.1	16,510	43.86	56.14	− 0.27	0.008	11,427	44.04	55.96	− 0.283	− 0.096	2647	45.18	54.82	− 0.112	0.232	1581	44.21	55.79	0.05	0.027	840	35.24	64.76	− 0.216	0.015
*P. novaeguineaensis*	KP638476.1	16,803	44.37	55.63	− 0.26	− 0.0	11,713	44.78	55.22	− 0.269	− 0.106	2647	45.11	54.89	− 0.111	0.232	1560	44.36	55.64	0.058	0.021	841	34.96	65.04	− 0.218	0.009
*P. novemradiatus*	MG680457.1	16,488	44.97	55.03	− 0.267	0.017	11,424	45.65	54.35	− 0.293	− 0.078	2638	45.38	54.62	− 0.099	0.209	1554	43.89	56.11	0.07	0.009	830	34.82	65.18	− 0.149	0.011
*P. schlosseri*	NC_030766.1	16,470	42.4	57.6	− 0.273	0.015	11,427	42.4	57.6	− 0.294	− 0.087	2551	44.61	55.39	− 0.116	0.22	1547	43.7	56.3	0.059	0.026	815	32.27	67.73	− 0.133	0.04
*P. serperaster*	KT965855.1	17,243	46.04	53.96	− 0.306	0.062	11,427	47.04	52.96	− 0.318	− 0.052	2630	46.65	53.35	− 0.121	0.253	1559	44.13	55.87	0.055	0.024	1586	39.28	60.72	− 0.39	0.157
*S. alcedo*	NC_018054.1	16,505	44.48	55.52	− 0.261	0.047	11,428	44.79	55.21	− 0.288	− 0.038	2640	44.85	55.15	− 0.083	0.229	1556	44.73	55.27	0.057	0.028	842	37.89	62.11	− 0.141	0.002
*S. gigas*	KT277705.1	16,717	45.22	54.78	− 0.277	0.035	11,427	45.84	54.16	− 0.298	− 0.069	2634	46.01	53.99	− 0.102	0.232	1561	45.1	54.9	0.045	0.039	1064	36.09	63.91	− 0.25	0.076
*S. histophorus*	JQ654459.1	16,496	44.13	55.87	− 0.269	0.014	11,428	44.32	55.68	− 0.291	− 0.079	2644	44.82	55.18	− 0.105	0.224	1561	44.2	55.8	0.046	0.04	830	37.59	62.41	− 0.147	− 0.031
*T. barbatus*	NC_018823.1	16,522	44.06	55.94	− 0.271	0.034	11,429	44.32	55.68	− 0.291	− 0.064	2646	44.97	55.03	− 0.101	0.232	1560	44.49	55.51	0.032	0.039	851	35.49	64.51	− 0.199	0.005
*T. bifasciatus*	JN244650.1	16,532	44.22	55.78	− 0.228	0.015	11,432	44.45	55.55	− 0.245	− 0.083	2665	45.33	54.67	− 0.075	0.224	1555	44.44	55.56	0.051	0.028	844	35.78	64.22	− 0.219	0.015

The base composition of the complete mitochondrial genome of *B. dussumieri* was determined to be A = 29.68%; C = 27.90%; T = 26.87% and G = 15.53%, respectively. In the mitogenome composition, we observed the GC and AT content, as well as the GC skew + and GC skew region. The overall GC and A + T content for the entire genome were calculated to be 43.43% and 56.54%, respectively, which were similar to the concatenated composition of the PCGs, tRNA and rRNA. Furthermore, the nucleotide composition of all mitochondrial genes exhibited a bias, with A + T content notably higher (56.54%) than the GC content (43.43%). This pattern of nucleotide composition is consistent with other mudskippers mitogenomes, and no significant differences were observed among them (Table 2). Furthermore, to access weather there were biased or not in the nucleotide composition, the GC and AT skew were measured for the whole mitogenome, as well as for the PCGs, ribosomal genes, and the control region. The GC and AT skew trends of all analyzed genes and control region are presented in (Table 2). Generally, most of the fish mitogenome present a clear bias in the nucleotide composition, which is the case of this study, where the GC skew was notably negative as most of the teleost fish^31–33^.

**Figure 3:**
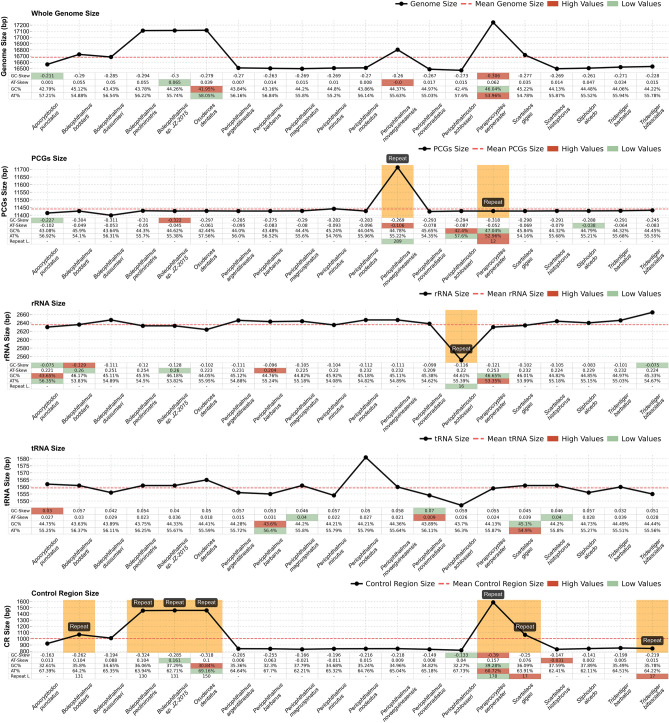
Composition and skewness. The brown bars represent the mitogenome repeat regions and the black line represents the size of the genes.

The GC and AT skew followed the same conventional preference of most mitogenomes, with AT skew positive, except for PCGs, and GC skew negative, except for tRNAs and CR, that varies from negative to positive in some mitogenomes. The AT skews of whole mitogenome ranged from − 0.0 (*Periophthalmus novaeguineaensis*) to 0.065 (*Boleophthalmus.*sp. JZ-2015), rRNAs 0.22 (*P. minutus*) to 0.254 (*Boleophthalmus pectinirostris*) whereas AT skew of the tRNAs range from 0.009 (*Periophthalmus novemradiatus*) to 0.039 (*Scartelaos giga*s), respectively (Fig. 3). These preferences in the sequenced mitogenomes are like other teleost mitogenomes^6–8^.

### PCGs, amino acid composition and codon usage

The PCG size of each mudskipper mitogenome ranged from 11,399 bp (*B. dussumieri*) to 11.713 bp (*P. novaeguineaensis)* (Supplementary Table S1). We compared seventeen mudskippers mitogenomes, and among these almost all PCG encoded in the H-strand, except for *ND6* that encoded in the L-strand, as typical of most vertebrate mitogenomes^10^. Overall, all PCGs showed the same configuration with ATG as start codon with exception of the *COI* gene that started with GTG codon. The preferences for codon termination of five PCGs (*NADH-1*, *NADH-2*, *COI*, *NADH-4L*, and *NADH-5*) were TAA and three (*ATP8*, *NADH-3* and *NADH-6*) terminated with TAG (Table 2). Some protein coding genes had an incomplete start and stop codon as in most fish mitogenomes^34,35^. For example, *NADH-4*, *COII*, *CYT B* gene, had an incomplete nucleotide T- whereas *ATP6* and *COIII* had incomplete TA- codon as stop codon (Table 1). These features are in accordance with most mudskipper fishes^36^. However, we found one unusual start and stop codon for most fishes (CCT/AAC) in *ATP8* gene of *Periophthalmus novemradiatus* species. The CCT codon, codes the Proaline amino acid whereas the AAC codes Asparagine amino acid.

Curiously, *P. novaeguineaensis* presented very peculiar codon preferences, totally different from others with only ten (ATG) as start codon, common GTG for COI, and three non-common codons (ATA for *ATP6* a, TTA for *ATP6* b, TTC and ACT for *NADH5-0* and *NADH5-1,* respectively). In contrast, the codon termination was also different from most mudskippers, presenting only four genes with TAA, as stop codon in the (*NADH-2*, *COI*, *NADH-4L* and *NADH-6*) genes and six different stop codons, AGG for (*NADH-1*), a common TAG stop codon for (*ATP8* and *NADH-3*), ACC for *ATP 6 a*, GTT for *ATP 6 b*, CTT and CTC for *NADH5-0* and *NADH5-1*, respectively.

**Figure 4:**
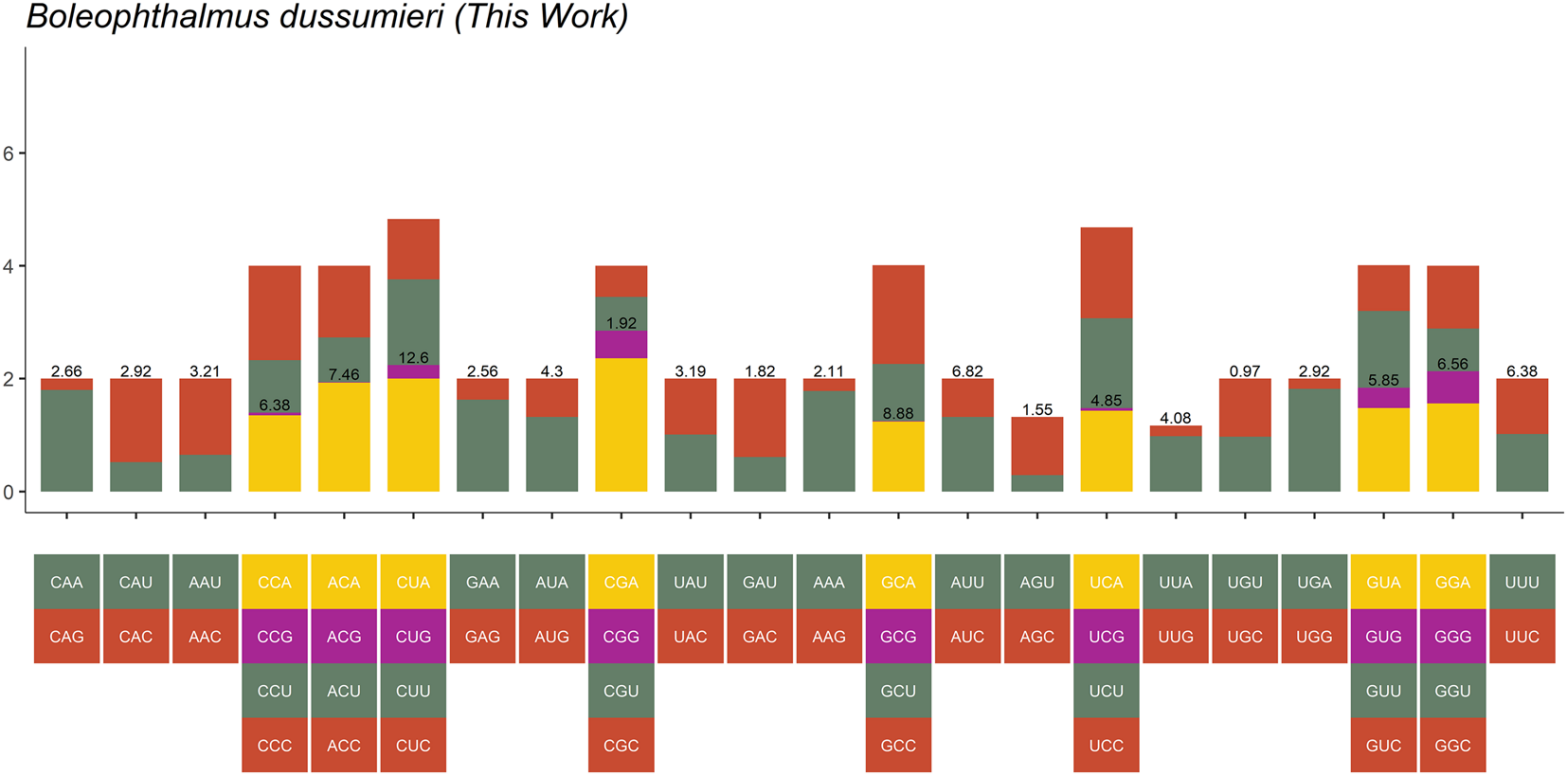
Relative synonymous codon usage in *Boleophthalmus dussumieri* mitogenome. Graphs from left to right represents Ala, Cys, Asp, Gln, Phe, Gly, His, Ile, Lys, Leu1, Leu2, Met, Asn, Pro, Gln, Arg, Ser1, Ser2, Thr, Val, Trp, Tvr.

The relative Synonyms codon usage (RSCU) is shown in (Fig. 4). The use of RSCU was biased for both two and six- fold degenerate codons. In overall, 5556 codons were analysed with exclusion of stop codons for *B. dussumieri* mitochondrial genome.

**Figure 5:**
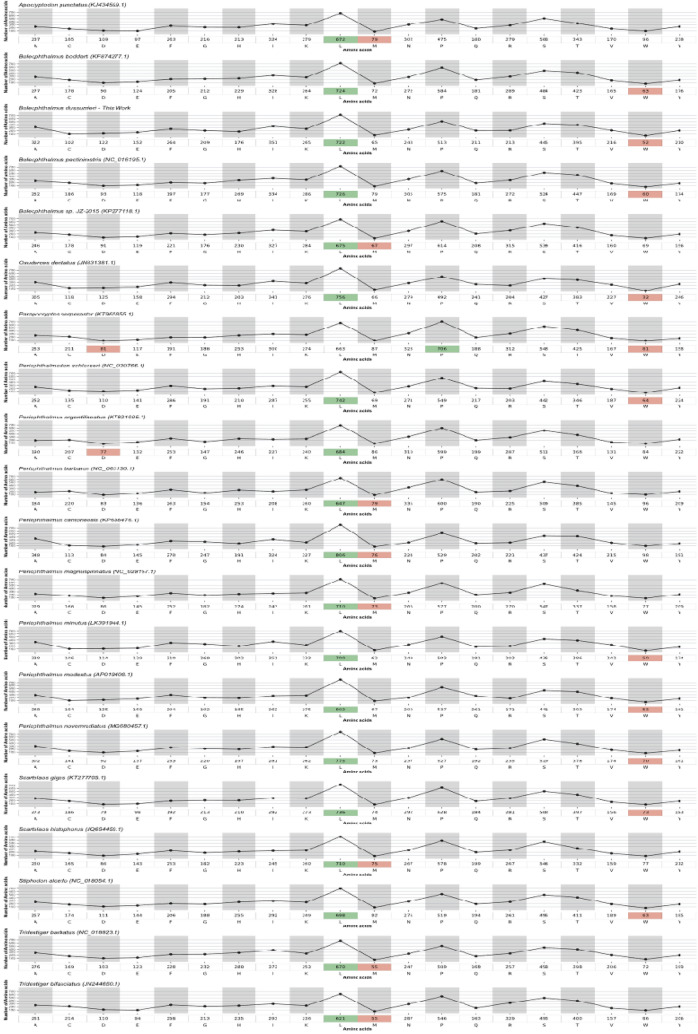
Amino acid composition of the *Boleophthalmus dussumieri* mitogenome.

The Codon degeneracy patterns indicates that most amino acids (14) Glu, Met, Tyr, Asp, Lys, Ile, Ser1, Leu2, Cys, Trp and Phe used a combination of two fold degenerated codon and eight amino acids (Pro, Thr, Leu1, Arg, Ala, Ser2, Val and Gly used four codon combination to encode with a duplication of Leucine and Serine, both with two fold degenerated codon (Fig. 4). These results were also observed in the literature^13,33^. The most used amino acid was Leu (13.75%), Pro (9.78%), Ser (8.48%) and Thr (7.51%) and the least used were Glu (2.89%), Trp (2.72%), Asp (2.32%), Arg (2.27%) and Cys (1.94), respectively (Fig. 5). The values appear to be similar, but the amino acid types are different from other studies^10,37–39^. All the detailed information about the amino acid composition and codon usage are in the Figs. 4 and 5.

### Ribosomal and transfer RNAs genes

Gene size showed similar values among all mudskippers. The *12S rRNAs* gene presented a size of 948 bp in a group that presented values between 947 and 956 bp, while for the *16S* rRNAs gene, the size of the genes varied from 1683 bp belonging to (*Parapocryptes serperaster*) to 1699 bp (*B. dussumieri*) (Supplementary Table S1). In general, the tRNAs genes were very conserved compared to some fish species^35,37,38^. We found 14 tRNAs out of 22 encoding the H-strand and eight (*trnA*, *trnC*, *trnE*, trnN, *trnP*, *trnQ*, *trnS2*, and *trnY*) in L-strand.

**Figure 6:**
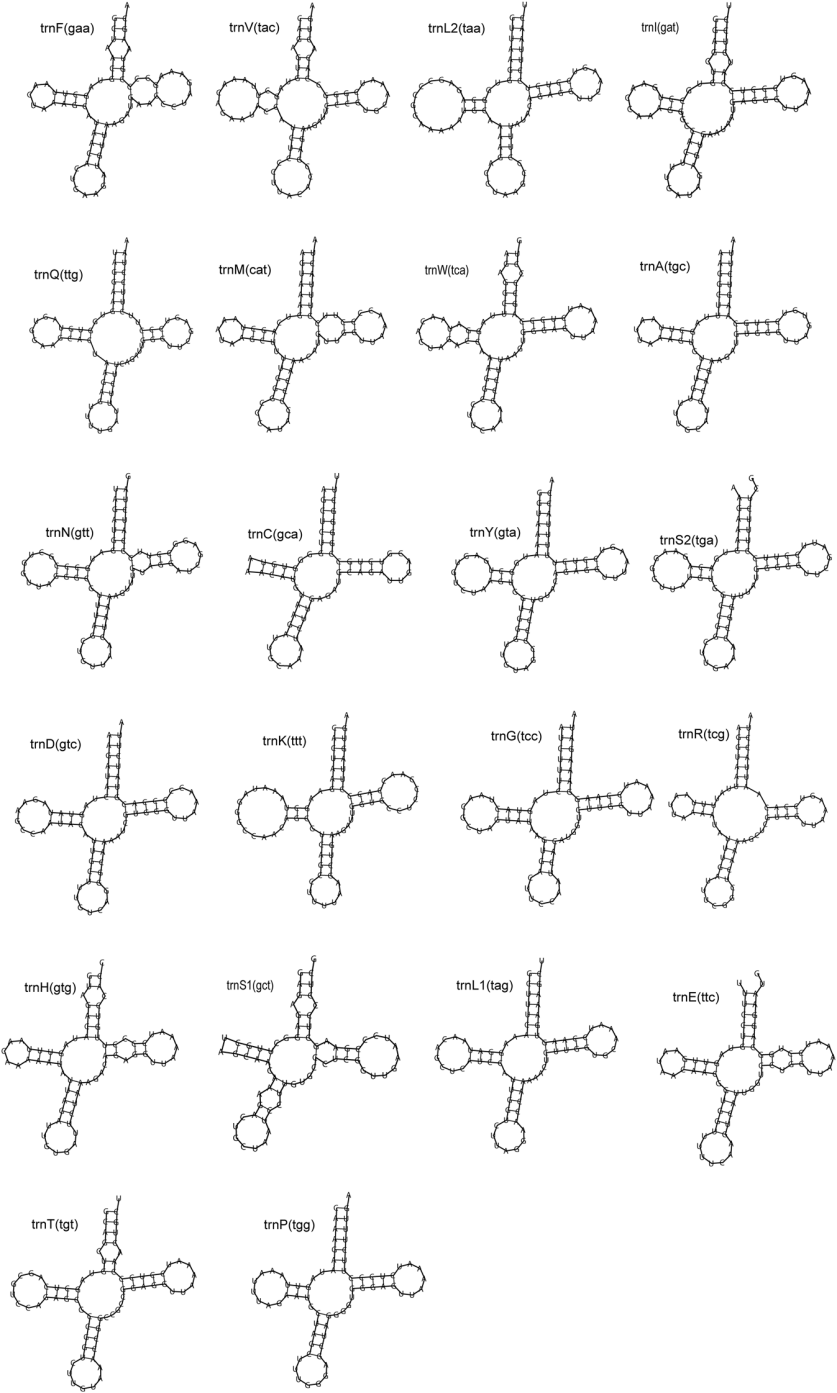
Secondary structures of tRNA in B. *dussumieri* mitogenome. The gene names of each tRNA are stated above each secondary structure.

The structure of almost all tRNAs were typical clover structure. Although most of them presented a secondary structure similar to other species with four arms and a central loop. Some of those tRNAs (trnM, trnS1, trnE, trnH, trnW, trnT, trnN, trnF, trnV, trnL) presented a variation in certain arms with presence of small loops (Fig. 6). The entire length of tRNA of the *B. dussumieri* was 1556 bp and the length of 22 individual tRNAs gene ranged from 65 to 76 bp. This value was between the length range of the other mudskippers analysed. (Supplementary Table S1).

### Gene rearrangement, repeated region, and concerted evolution

The organization of almost all vertebrate animals generally have their mitogenome structurally conserved with one set gene copy, no intron or long intergenic spacer and single CR^40,41^. Nevertheless, gene rearrangement in mitogenome of vertebrates are deeply explained using different models including shuffling, translocation, and inversion^42^, but most common is duplication and deletion model approaches^43,44^. The CR of this study followed typical rearrangement located between the *tRNA-Pro* and *tRNA-Phe* gene, as most vertebrates mitogenomes^10,45^.

We analysed seventeen mudskippers’ species and nine of them presented duplication in their mitogenome specially in the control region. The duplication was found in three different regions, most of them, in a non-coding gene, the control region (*Boleophtalmus boddarti*, *B. pectinirostris, Boleophthalmus sp* JZ-2015, O*xuderces dentatus*, *S. gigas*); three in the PCGs, *ATP6*, *NADH-5 gene* (*P. novaeguineaensis*)*, ATP8* (*P. novemradiatus* and *P. minutus*); and one ribosomal gene, the *12S rRNA* gene duplication in the *Periophthalmodon schlosseri*. Our study shows two species with the OH region duplicated, *P. serperaster* with four OH region, with the sequence length ranging from 78 to 320 bp, and *S. gigas* with one duplication involving 31 bp and 316 bp, respectively (Table 4 and Fig. 3). Furthermore, our results also presented one species (*P. serperaster*) with both CR duplication and one coding-region duplication, the *NADH-1* gene.

So how can these duplication mechanisms in mudskipper be explained? Several models are suggested to be involved in fish mitogenome rearrangements^10^. In this study the conformity of the gene did not show any gene reverse or abnormal gene exchange and because this model is so rare in fish mitogenome, we consider that is not appropriate to explain the rearrangement in the mudskipper mitogenome^44^. The second most studied model for mitogenome rearrangements is the TDNL and DRRL models. In the majority of cases, these models offer explanations for instances of gene clustering based on their site of encoding. (L- or H-chain coding)^46,47^.

These models are not suitable to explain the rearrangements or the duplication observed in the present study. Therefore, we consider that the model that best suits the redundant rearrangements observed in mudskippers is the tandem duplication-random model (TDRL), mainly because it is related to the repetitions and duplications that are found in some portions of the mitogenome^48,49^. For example, further we explain the rearrangement found in the *Boleophthalmus* group, where repetition and duplication events of the motif region were very frequent, associated with the presence of intergenic spacer and incorrect initiation or termination of certain genes, followed by random deletions of part of these fragments specially in the control region^50^.

### Gene duplication in the *Boleophthalmus* genus

The genus *Boleopthalmus* was represented by four species, *B. dussumieri* (this study), *B. boddarti, Boleopthalmus pectinirostris* and *Boleophthalmus*. sp. The first was considered the basal species and thus being the first to be analysed and further identified the motif fragment in their mitogenome, however no duplication was found. On the other hand, the *B. boddarti* presented 131 bp of the motif sequence, which was duplicated 2.4 times, whereas the other two species (*B. pectinirostris* and *Boleophthalmus* sp.) had this region repeated 5 times, meaning that there was a systematic increasing of the region (Fig. 3). This phenomenon is common in most fish mitogenomes^34^, however there are other different gene organization the fish mitochondrial genome^10^. All the repeats were found in the 5′–3′ directions. The alignment of the motif region of all *Boleophthalmus* was clearly similar showing that this duplication evolved in concert^51^. In addition, studies show that, when the motif region is considered homologues, the phenomena is therefore considered concerted evolution^52,53^.

### Intergenic spacers, overlapping and evidence of TDRL/DMNL models

Intergenic spacers (IGS) are non-coding regions that are found between genes and are typically found in Metazoa and most vertebrate animals and in most cases serve as a transcription promotor mitogenomes^50,54^ and, they have influence on the growth rate of some invertebrate animals^55^. They are important signal for evolutionary studies and even species delimitation^56,57^. Besides IGS event, there is also segments of gene overlapping in mitochondrial genome^45^.

In the newly sequenced mitogenome of *B. dussumieri* there were 17 intergenic spacer regions with a total sequence length of 899 bp (Table 3). Four of these regions were considered long IGS with the sequence length ranging from 14 bp, located between *COX III* and *tRNA-Gly*, 51 bp between *tRNA-Val* and *r16S*; 117 bp between *ATP6*/*COX III* on the H-strand and one longest IGS of 495 bp located between *tRNA-Pro*/OH on the L-strand. Large intergenic spacer has been reported in many vertebrate mitogenomes^58,59^. Besides those, all of IGS had a length ranging from (1–8 bp). To explain this phenomenon, three main evolutionary mechanisms of IGS origin in mitogenomes have been discussed in previous studies, the tandem duplication/random loss (TDRL) model and slipped-strand mispairing and the dimer-mitogenome and nonrandom loss (DMNL) when there is similarity in mitogenome rearrangements^10,60,61^.

**Table 3:** Intergenic spacer and overlap regions.

Species	Total length (IGS) bp	IGS	Loongest IGS	Region with the Longest IGS	Overlapping	Nr of bp involved	Conserved region
*Apocryptodon punctatus*	465	16	162	(OH)	7	(− 1 to − 7)	16
*Boleophthalmus dussumieri*	899	17	495	(tRNA-Pro/OH)	7	(− 1 to − 7)	15
*Boleophthalmus boddardti*	832	17	425	(tRNA-Pro/OH)	9	(− 1 to − 7)	16
*Boleophthalmus *sp.* JZ-2015*	1207	14	706	(OH)	9	(− 1 to − 7)	15
*Boleophthalmus pectinirostris*	1182	16	422	(tRNA-Pro/OH)	9	(− 1 to − 7)	17
*Paraprocryptes Serperaster*	887	17	516	(tRNA-Pro/OH)	9	(− 1 to − 7)	17
*Periophthalmus minutus*	439	15	317	(tRNA-Pro/OH)	10	(− 1 to − 20)	15
*Periophthalmus barbarus*	437	14	320	(tRNA-Pro/OH)	10	(− 1 to − 7)	15
*Periophthalmus modestus*	441	14	321	(tRNA-Pro/OH)	9	(− 1 to − 7)	16
*Periophthalmus magnuspinatus*	431	13	307	(tRNA-Pro/OH)	9	(− 1 to − 7)	17
*Periophthalmus novemradiatus*	426	16	308	(tRNA-Pro/OH)	10	(− 1 to − 20)	14
*Periophthalmus Cantonensis*	886	17	385	tRNA Leu1/ ND5_0	7	(− 1 to − 7)	15
*Periophthalmus argentilineatus*	441	13	321	(tRNA-Pro/OH)	9	(− 1 to − 7)	16
*Periophthalmodon schlosseri*	418	13	301	(tRNA-Pro/OH)	9	(-1 to − 7)	17
*Oxuderces dentatus*	1195	13	614	(tRNA-Pro/OH)	10	(− 1 to − 7)	15
*Scartelaos gigas*	773	13	522	(tRNA-Pro/OH)	12	(− 1 to − 7)	15

To understand how rearrangements in intergenic spacing occurred in mudskippers, it is important to observe the location of gene spacing. In this study, the gene spacing with high values occurred in the same position (tRNA-Pro/OH) except for *Periophthalmus cantonensis* which had its spacing in the tRNA Leu1/ND5_0 gene which probably did not follow the same rearrangement mechanism. Another important aspect tobe observed is the variation in the sizes of base pairs involved. This phenomenon is common in vertebrate mitogenomes^32^. Looking at these variations in the mitogenome rearrangements, it raises questions about how these phenomena occur and why there are similarities in its positions? The DMNL model supports dimerism in the vertebrate mitogenome, a fact that may condition this conformity. In fact, dimeric conformity in the fish mitogenome have already been observed^60^. Anguilliformes fish is a group is fish where this scenario was deeply elucidated.

In addition to IGSs, seven overlapping sequences were also observed in *B. dussumieri* mitogenome, ranging from (1–7 bp) (Table 2). The total length of the sequence was (24 bp) that was divided in to two main region *ATP8*/*ATP6* (7 bp) and *NADH-4L*/*NADH-4* (7 bp), both on the H-strand. These two overlapping regions, both being 7 bp of length, are very common in fish mitogenomes, including mudskippers (Table 3)^31,62^.

The comparative analysis of the IGS and overlapping regions presented several variations in numbers, length, and locations within mudskippers. The number of IGS regions ranged from 13 to 17 with the total length varying from 418 (*P. schloseri*) to 1207 bp (*Boleophthalmus *sp.) which have the largest IGS (706 bp) located in the OH region (Table 3). Most of large IGS were located between *tRNA-Pro* and OH region, but only one (385 bp), belonging to *P.* n*ovaeguineaensis*, was located between *tRNA-Leu1* and *NADH5_0*.

In overall, the overlapping length varied from 1 to 7 bp in most mitogenomes. However, two species of the genus *Periophthalmus* presented unusual overlapping, involving 20 bp (*P. minutus* and *P. novaeguineaensiss*). This may be due to the split/duplication of the ATP8 gene in these species. The study also presents some peculiarities involving the IGS of the OH regions. We found large IGS of the OH region ranging from 61 bp (*Periophthalmus modestus*), to 185 bp (*B. dussumieri*), and very large varying from 314 bp (*B. boddarti*) to 701 bp (*B. pectinirostris*) (Table 3).

### Phylogenetic analysis

**Figure 7:**
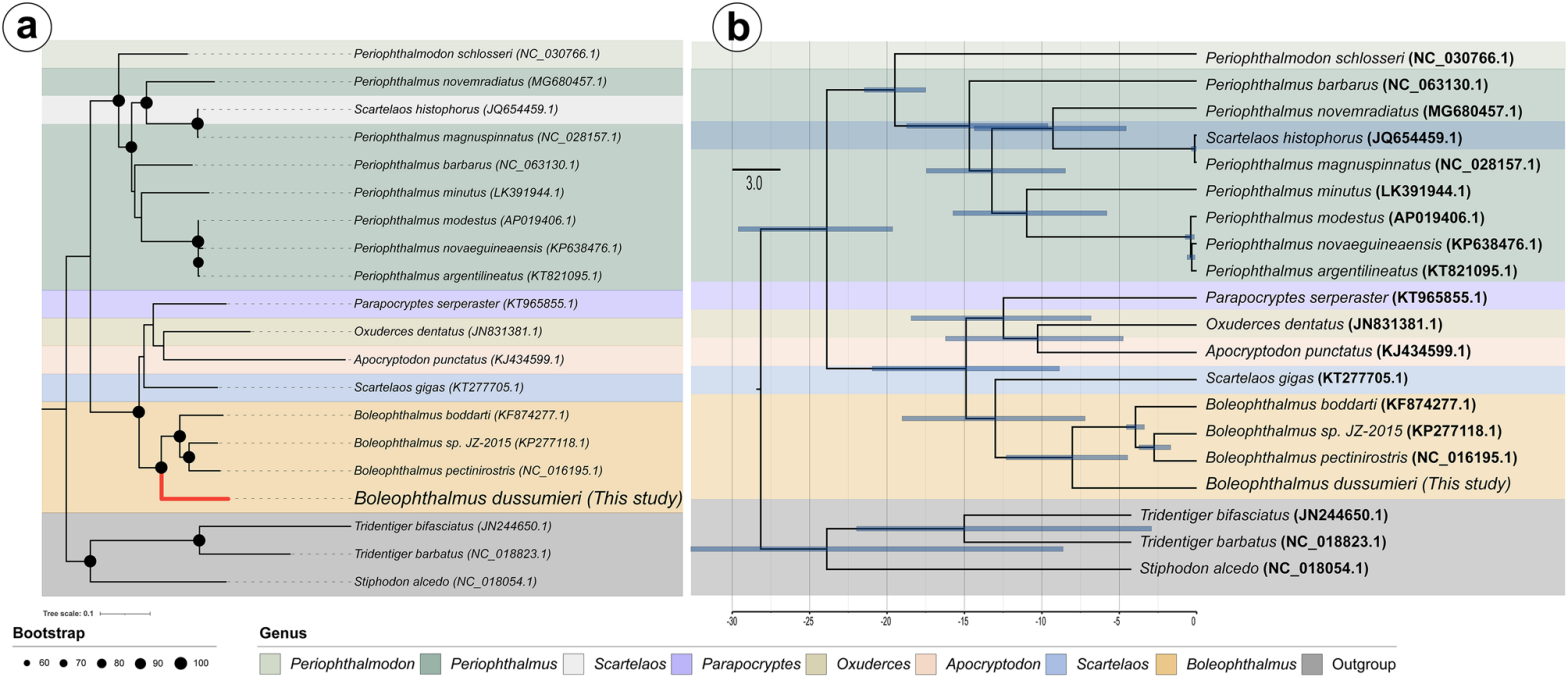
(**A**) Phylogenetic relationships mudskippers species based on the maximum-likelihood methods from concatenated nucleotide sequences of the 13 PCGs regions. (**B**) Mean divergence times were estimated using a relaxed molecular clock model on a subset of mitochondrial genes. The colors indicate the genus group.

The phylogenetic relationship within mudskippers were determined using a combination of 13 protein-coding genes (PCGs) and 2 ribosomal genes from a total of 17 mudskipper species. Additionally, three species of gobies (*Tridentiger bifasciatus, Tridentiger barbatus* and *Stiphodon alcedo*) were included as outgroups. The evolutionary tree was constructed using the ML method. The resulting phylogenetic tree for the mudskippers positioned them as monophyletic group with a high support value (ML bootstrap = 100) (Fig. 7A). Several other studies have provided evidence for the monophyly of mudskippers using complete mitogenome analysis and analysis of the *COI*^63^. These studies have identified two major groups within the mudskippers. The first consists of all *Periophthalmus* species, which form a sister group of *Periotphthalmodon*, with high support values (> 90). The second group is comprised of two subclades. One subclade includes *Boleophthalmus*, which is sister group of *Scatelaos*, with a support value of >  = 90. The other subclade consists of *Apocryptodon, Paraprocryptes,* and *Oxuderces*, with bootstrap value > 50. *Scartelaos* is grouped together with the *Periophthalmus* clade. The *Periophthalmus* clade is considered monophyletic and there is high similarity (> 99%) of *Scartelaos histophorus* with *Periophthalmus magnuspinnatus* (GenBank accession numbers KT284931.1, KT357639.1), so the presence of *S. histophorus* (accession number JQ654459.1) in the *Periophthalmus* clade should be considered misidentification^64^. Therefore, the specie revalidation in the Genbank should be considered to avoid further taxonomic problems.

The topology position of *P. barbarus* presented a different clustering in both trees. In the ML tree the taxon clustered together with *P. minutus*, *P. modestus*, *P. novaeguineaensis* and *P. argentilineatus* as a sister group, whereas in the IB the species was positioned out of the sister genus. The results presented in this study brings a clarification in relation to the separations of the groups within mudskippers.

### Estimation of divergence times

In our phylogenetic analyses, the target species of the study, *B. dussumieri*, was correlated with other species of its genus and the genus Periophthalmus. Both the BEAST and ML analyses showed similar topologies in our simulations (Fig. 7A, B). The TMRCA estimated by BEAST demonstrated that the diversification of mudskippers from the Gobiidae family occurred approximately 28.72 million years ago (Ma) (Fig. 7B). Within the mudskippers, the genera Periophthalmus and Periophthalmodon were the first to diverge and diversify, dating back to around 19.52 Ma in the Early Miocene (Fig. 7B). This suggests that these genera represent the basal lineages within terrestrial gobies. The divergence between the clades of Boleophthalmus and Periophthalmus occurred around 24.42 Ma in the Early Miocene (Fig. 7B). *Boleophthalmus dussumieri* and its sister species within the genus are estimated to have diverged around 8.22 Ma during the Late Miocene (Fig. 7B). Comparing the divergence time of *B. dussumieri* with another sister genus, the estimated times were roughly 14.88 Ma for Exuderces, Apocryptodon, and Paraprocryptes, and 12.82 Ma for Scartelaos. These values are consistent with those recovered from other studies on closely related families, which are sister families of Mudskippers (Oxudercidae), including the subfamilies Amblyopinae Günther, 1861, Gobionellinae Bleeker, 1874, and Sicydiinae T.N. Gill, 1860^65,66^. The minimum and maximum ages, with 95% Highest Posterior Density (HPD) for individual and group node ages, are shown in Table 4.

**Table 4:** Confidence intervals of the diverge time scales.

Taxa	Min and Max confidence Intervals (95% HPD)
Tridentiger genus*/Stiphodon alcedo*	(8.62–33.66)
Tridentiger genus	(2.92–21.96)
Periophthalmus genus/Boleophthalmus genus	(19.64–29.60)
Periophtahlmus genus/Periophthalmodon genus	(17.5–21.46)
*P. barbarus/*other Periophthalmus	(9.61–18.72)
*P. modestus; novaeguineaensis; argentilineatus/P. magnuspinatus; novemradiatus*	(8.48–17.46)
*P. minutus/P. modestus; novaeguineaensis; argentilineatus*	(5.8–15.74)
*P. magnuspinatus/P. novemradiatus*	(4.55–14.36)
Boleopthalmus genus*; Scartelos gigas/P. serperaster/A. punctatus/O. dentatus*	(8.87–20.96)
Boleopthalmus genus*/Scartelos gigas*	(7.21–19.02)
*Boleopthalmus dussumieri/*other Boleophthalmus species	(4.46–12.29)
*B.boddarti/Boleophthalmus sp_JZ2015/B. pectinirostris*	(3.37–4.54)
*Boleophthalmus sp_JZ2015/B. pectinirostris*	(1.68–3.72)
*P. serperaster/A.punctatus/O. dentatus*	(6.82–18.44)
*A. punctatus/O. dentatus*	(4.74–16.21)

## Conclusion

In this study we present the complete mitochondrial genome of the mudskipper species *B. dusumieri* with 16,685 bp of length and the first comparative mitogenome gathering all available mudskipper mitogenomes**.** Generally, the organization followed almost the same configuration as most fish mitogenomes. However, we found different peculiarities regarding gene rearrangement, long intergenic spacer, and duplication of gene fragments mostly on the CR but also in a coding gene and one ribosomal gene in the analysed mitogenomes. The phylogenetic reconstruction using 13 concatenated PCG positioned all mudskipper species as monophyletic group within the family Oxudercidae. The phylogenetic clustering recovered in this study are in accordance with other previous study. The time scale estimation demonstrated that within mudskippers, the *Periophthalmus* genus was the first to diverge in early Miocene and thus considered basal group. The complete mitogenome generated in this study is a source of genetic information for further molecular studies including conservation strategies.

## Material and methods

### Sample and DNA extraction

**Figure 8:**
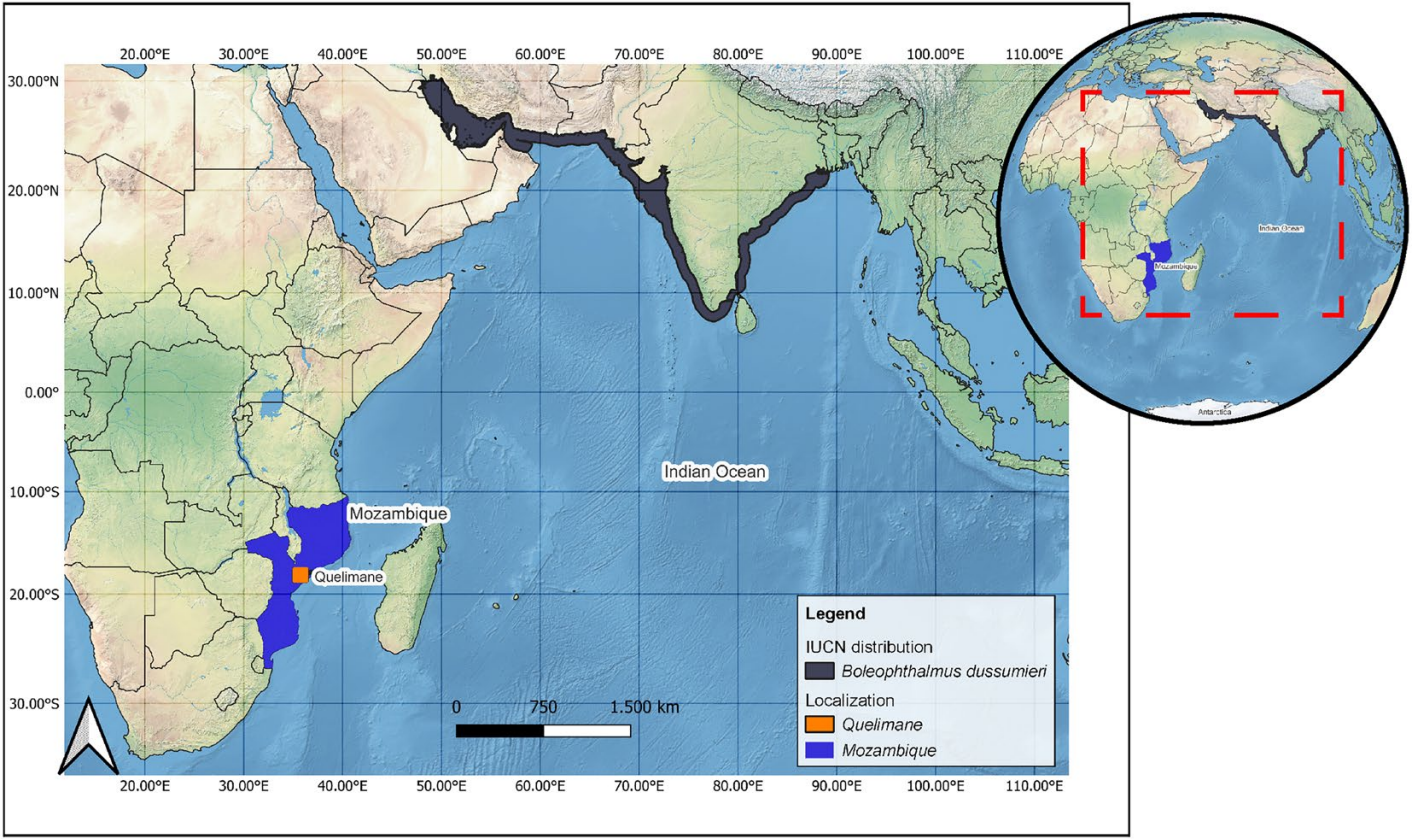
Sampling sites for *B. dussumieri* mudskipper specimens in the Bons Sinais estuary, Quelimane, Mozambique. The Shapes used were obtained from the Natural Earth website (https://www.naturalearthdata.com/, dataset available at 1:10 m, 1:50 m, and 1:110 million scales), and the maps were created using the QGIS software (https://qgis.org/en/site/, V 3.32).

The mudskipper specimens used in this study were collected from the Bons Sinais estuary in Quelimane, Mozambique (Fig. 8). These samples belong to the *B. dussumieri*, which was recently identified for the first time on the cost of Mozambique^67,68^, an area that its occurrence is not quoted by IUCN. Upon collection, the fresh specimens were immediately placed in 96% ethanol and stored at – 20 °C. Voucher specimens were preserved in 10% formaldehyde and deposited at the Maputo Natural History Museum with the code MHNM.PIS.2021.0398.

Muscle tissue was extracted from specimens and taken to the Evolution Laboratory at the Federal University of Pará, Brazil, for molecular analysis. One specimen was chosen for further analysis. Genomic DNA was extracted from the muscle tissue using a Wizard Genomic DNA Purification Kit (Promega Corporation, Madison, WI—USA), following the manufacturer’s protocol. The purity and concentration of the DNA were assess using a spectrophotometer, and successful extraction was confirmed through 1% agarose gel electrophoresis. The DNA samples were then stored at – 20 °C.

### Ethics declarations

All biological material used in this research came from an artisanal fishery and were exclusively for general fishery purpose. No live specimen was kept in captivity or manipulated. Therefore, no ethical approval was necessary.

### PCR amplification and sequencing

Firstly, nineteen primers (Supplementary Table S2) were designed using Primer3 (https://academic.oup.com/nar/article/40/15/e115/1223759?login=true) implemented in GENEIOUS software based on the conserved regions of all available complete mitochondrial genome from the family, taking into consideration the closest species within the genus. To optimize the amplification of uncovered fragments, specific primers were redesigned.

The PCRs were run in a final volume of 15 μl containing 2.5 μl of dNTPs (1.25 mM), 1.5 μl of 10 × buffer solution, 0.7 μl of MgCl_2_ (50 mM), 0.5 μl of each primer (10 pmol/μl), 1.0 μl of total genomic DNA (100 ng/μl), 0.2 μl of Taq DNA polymerase (5 U/μl) and pure water to complete the final volume of the reaction following previous studies^67,68^. The amplification protocol consisted of initial denaturation at 94 °C for 3 min, followed by 35 cycles of denaturation at 94 °C for 30 s, annealing at 52–65 °C for 40 s, then extension at 72 °C for 45 s, followed by a final extension at 72 °C for 5 min. All positive reactions were sequenced using the Sanger method with the same primers used to amplify the fragment, with a BigDye Terminator v3.1 Cycle Sequencing Kit (Applied Biosystems), according to the manufacturer's instructions. Electrophoresis was performed on ABI 3500 XL (Thermo Fisher). Negative controls were included in all PCR reactions to confirm the absence of contaminants.

### Sequence assembly, annotation, and analysis

After sequencing the fragments, the Bowtie tool, implemented in Geneious 9, was used to map the generated sequences against the reference and generate the consensus sequence. For mapping the consensus sequence, the species *B. boddarti* was used as a reference. The complete mitogenome was annotated using the Mitos 2 (http://mitos.bioinf.uni-leipzig.de)^69^ and later confirmed in MitosZ 3.6 (https://github.com/linzhi2013/MitoZ) with reference to Metazoa 63. The analysis of the repetition identification regions was performed using the finder tinder repeat tool (https://academic.oup.com/nar/article/27/2/573/1061099?login=true). The base composition, amino acid calculation as well as relative synonymous codon usage (RSCU) of PCGs were estimated using MEGA 11^70^.

The base composition values (AT- and GC-skews) were calculated using the following formulas: AT-skew = (A−T)/(A + T) and GC-skew = (G−C)/(G + C). The genetic order was calculated using the Phylosuite^71^.

### Phylogenetic analysis

To investigate the phylogenetic relationship among all mudskippers, a phylogenetic tree was constructed using a final dataset of 20 goby species. This dataset was based on the combinations of 13 PCG and two RNAs genes.

To accomplish that, we performed Maximum Likelihood (ML) analysis in the IQTree v 2.2.0 program. The best partitioning scheme for the database were adopted considering the position of the codons of each gene, as well as the evolutionary models for the respective partitioning scheme. Subsequently the ML analysis was run with 1000 bootstrap pseudo replicates.

### Divergence time analysis

Divergence time analyses were conducted using BEAST v.1.10^72^. The uncorrelated relaxed clock^73^ was employed, and the Yule process served as the prior model for the tree^74^. Calibration points were incorporated to estimate divergence time, including the separation between *B. boddarti* and *B. pectinirostris* being calibrated to 3.8 Ma, based on Chen et al.^63^, which used references from the study conducted by Mukai et al.^75^, considering the mutation rate (1.95 ± 0.17) % per million years per lineage per site of the Rhinogobius ND5 gene, while the separation in the *Periophthalmus* genus was calibrated to 20.09 Ma based on the calibration proposed by^76^ that used approximate (2 μ) paired molecular clock of 3.8%/myr for the ND5 gene from related gobies (Rhinogobius).

The analysis was run for 100 million generations, with log parameters recorded every 100 registered at each 5000 generations. Trees were summarized in TreeAnnotator v.1.8.4^72^. A burn-in of 20% was used and the run was considered satisfactory when all ESS values checked in Tracer v.1.6^77^ were equal to or larger than 200.

### Supplementary Information

Supplementary Tables.

## Data Availability

Sequences have been submitted to NCBI and will be available after acceptance. Other data supporting the findings of the present study are cited accordingly in the manuscript and in its Supplementary Information files, or from the corresponding authors upon request.
