# Supplementary material for: Comparative mitochondrial genome brings insights to slight variation in gene proportion and large intergenic spacer and phylogenetic relationship of mudskipper species
Source: Sci Rep. 2024 Feb 9;14:3358. doi: 10.1038/s41598-024-52979-4 (PMC10858209; doi:10.1038/s41598-024-52979-4)
Supplement: Supplementary file 1 — Supplementary Tables. [file 41598_2024_52979_MOESM1_ESM.pdf]

Comparative mitochondrial genome brings insights to slight variation in gene proportion and large intergenic spacer and phylogenetic relationship of mudskipper species.

Valdemiro Muhala[1,2*]; Aurycéia Guimarães-Costa[1]; Adam Rick Bessa-Silva[1];Luan Rabelo[1]; Jeferson Carneiro[1] ; Isadola Eusébio Macate[1]; Luciana Watanabe[1]; Oscar David Balcázar[1]; Grazielle Evangelista Gomes[3] , Marcelo Vallinoto[1,4] and Iracilda Sampaio[1]

1.Universidade Federal do Pará, Instituto de Estudos Costeiros, Laboratório de Evolução Bragança Pará, Brazil

2.Instituto Superior Politécnico de Gaza, Divisão de Agricultura, Chókwè 1204, Mozambique

3.Universidade Federal do Pará, Instituto de Estudos Costeiros, Laboratório de Genetica Aplicada ,Bragança, Pará, Brazil
4.Centro de Investigação em Biodiversidade e Recursos Genéticos, Laboratório Associado, Campus agrário de Vairão, Universidade do Porto, Vairão, Portugal.

***Corresponding author:** *Valdemiro dos Pereira Carlos Muhala*
Email: valdemiro.muhala@ispg.ac.mz; Tel: +5591988060183

Nome	tRNA-Phe	12S	tRNA-Val	16S	tRNA-Leu	NADH-1	tRNA-Ile	tRNA-Gln	tRNA-Met	NADH-2	tRNA-Trp	tRNA-Ala	tRNA-Asn	tRNA-Cys	tRNA-Tyr	COI	tRNA-Ser	tRNA-Asp	COII
A. punctatus	68	952	79	1678	75	975	70	70	69	1047	71	69	73	67	69	1551	71	72	691
B. boddarti	68	947	72	1689	75	975	70	71	69	1047	71	69	73	65	71	1554	71	72	691
B. dussumieri	68	948	72	1699	75	975	70	71	69	1047	71	69	73	65	71	1554	71	72	691
B. pectinirostris	68	947	72	1686	75	975	70	71	69	1047	71	69	73	65	71	1554	71	72	691
B. sp. JZ-2015	68	947	72	1686	75	975	70	71	69	1047	71	69	73	65	71	1554	71	72	691
O. dentatus	68	948	80	1676	75	975	70	71	69	1047	72	69	73	64	71	1554	71	72	691
P. barbarus	69	949	72	1697	76	975	70	71	70	1047	72	69	73	67	71	1554	71	73	691
P. cantonensis	68	956	72	1691	75	976	70	71	69	1047	71	69	73	65	70	1554	71	72	691
P. magnuspinnatus	68	956	72	1688	75	975	70	71	69	1047	71	69	73	65	71	1554	71	72	691
P. minutus	68	952	72	1683	75	975	70	71	69	1047	71	69	73	65	71	1554	71	72	699
P. modestus	68	956	72	1691	75	975	70	71	69	1047	98	69	73	65	70	1554	71	72	691
P. schlosseri	68	947	72	1604	75	975	70	71	69	1047	71	69	73	65	71	1554	71	65	691
P. serperaster	68	947	72	1683	75	975	70	71	70	1047	71	69	73	65	71	1554	71	72	691
P.argentilineatus	68	955	72	1691	75	975	69	71	68	1047	71	69	73	65	70	1554	71	72	691
P.novemradiatus	68	954	72	1684	75	975	70	71	69	1047	71	69	73	65	70	1554	71	72	691

Nome	tRNA-Lys	ATP-8	ATP-6	COIII	tRNA-Gly	NADH-3	tRNA-Arg	NADH-4L	NADH-4	tRNA-His	tRNA-Ser	tRNA-Leu	NADH-5	NADH-6	tRNA-Glu	CYTB	tRNA-Thr	tRNA-Pro	dloop
A. punctatus	76	165	678	784	72	349	69	297	1381	69	68	73	1836	519	69	1141	72	71	923
B. boddarti	76	165	682	784	72	349	69	297	1381	69	74	73	1839	522	69	1141	72	70	1067
B. dussumieri	76	165	717	721	72	349	69	297	1381	69	68	73	1839	522	69	1141	73	70	1010
B. pectinirostris	76	165	684	784	72	349	69	297	1381	69	74	73	1839	522	69	1141	72	70	1453
B. sp. JZ-2015	76	165	682	784	72	349	69	297	1381	69	74	73	1839	522	69	1141	72	70	1456
O. dentatus	76	165	683	784	72	349	69	297	1381	69	68	73	1839	522	69	1141	73	71	1456
P. barbarus	75	165	684	784	71	349	69	297	1381	69	68	73	1839	522	69	1141	72	70	839
P. cantonensis	76	165	677	784	72	349	69	297	1381	69	68	73	2129	522	69	1141	72	76	841
P. magnuspinnatus	76	165	683	784	72	349	69	297	1381	69	68	73	1839	522	69	1141	72	76	831
P. minutus	76	165	682	784	72	351	69	297	1386	69	68	73	1839	522	68	1141	72	70	839
P. modestus	76	165	682	784	72	349	69	297	1381	69	68	73	1839	522	69	1141	72	70	840
P. schlosseri	76	165	682	784	72	349	69	297	1381	69	67	73	1839	522	69	1141	72	70	815
P. serperaster	76	165	682	784	72	349	69	297	1381	69	71	73	1839	522	69	1141	72	70	1586
P.argentilineatus	76	165	682	784	72	349	69	297	1381	69	72	73	1839	522	69	1141	72	70	840
P.novemradiatus	76	165	682	784	72	349	69	297	1378	69	67	73	1839	522	69	1141	72	71	830

S. alcedo	68	950	72	1690	76	975	70	71	69	1047	71	69	73	66	71	1554	71	72	691	75	165	683	784	72	349	69	297	1381	69	68	73	1839	522	69	1141	72	70	842
S. gigas	68	948	71	1686	75	975	70	71	70	1046	71	69	73	65	71	1554	71	72	691	76	165	683	784	72	349	69	297	1381	69	74	73	1839	522	69	1141	72	70	1064
S. histophorus	68	956	72	1688	75	975	70	71	69	1047	71	69	73	65	71	1554	71	72	691	76	165	683	784	72	349	69	297	1381	69	68	73	1839	522	69	1141	72	76	830
T. barbatus	68	953	72	1690	75	975	70	71	69	1047	71	69	73	65	71	1554	71	72	691	76	165	682	784	72	349	69	297	1381	69	68	73	1839	522	69	1141	72	70	851
T. bifasciatus	69	954	72	1711	75	975	69	71	69	1047	71	69	73	67	71	1560	71	72	691	75	165	681	784	71	349	69	297	1381	69	68	73	1839	522	69	1141	72	70	844

Suplementar Table 1. Gene size of all Mudskippers species and three goby fishes

**Primer**	**Nucleotide sequence (5'- 3')**	**MT**
F1	GCATAACACTGAAGATGTTAAGA	54
R1	CAGTGTGGCCCGATTTGCACGGG	
F2	GAAACTGGCCCTGAAGCGCGCACAC	53
R2	CCTTGGGCACACCTGTGTGGGGTTAACG	
F3	ACTGCTAACATGAGTAATAAGGG	54
R3	GGAGAGGACTTGAACCTCTGTT	
F4	AGTACGAAAGGACCGAAAAGAG	54
R4	CAAAGTGGCCCTTTGTTTCAGGCAC	
F5	GCCCTATTTTTCCTTGCAGAATATGC	53
R5	CCTGTTAATGGGGGRAGGCCTCCTA	
F6	CAACTGCAGCATTCTTAACCCTAAAAA	60
R6	GGTATTACTATAAAGAAAATTATTACAAA	
F7	GGCACCCTTTATCTTGTATTTGGTGCC	53,4
R7	CCTGTTGGGATGGCAATAATTATTGTG	
F8	TTGTTGCCTACTATGCAGGWAAAAAAG	53,4
R8	TCTTCTATTACAGGTGATGCTGCATCT	
F9	CCCTTCTGTCACTTTCTTAATAAGAT	60
R9	TTGCTATCATGYTCAGGWTCAGGGG	
F10	CCCAACCACCCTTAGTGACATGCCCC	
F11	GGCCCACCAAGCACATGCATACCAC	58,1
R11	GGGTCAAAKCCACATTCATAGGGYGA	
F12	GGTCTTGGTTAAAATCCAAGGAAAG	58
R12	ATCCGTTGRCGGTTAARTGGCTCA	
F13	GACCYACAGCCCTYGCCCACAGCC	62,5
R13	GGAGGGCRAGGTTTGCTAGGCTGGC	
F14	CYAACTATGAACGRACCCACACCCG	58,1
R14	CCYACRCCTTCTCAMCCAATAAAG	
F15	GTTGCYCTRTATGTARCCTGGTC	56
R15	GRTGTGTTTAGGGCTTCAATAATTG	
F16	AGGAGGRATACACCACCTTGCYCC	57,6
R16	GTTGTGTTTGCTTATTCTYCTGCT	
R17	GGGGATTTTGTCTGCATCTGAGT	
F18	GGYTTTTCAGTTGACAATGCAAC	53,6
R18	GGTGGGAGTTAAAATCTCCCTTCTT	
F19	CCGGTCTTGTAAACCGGCCGCCGGA	59,9
R19	GGTTGWGCCTAATYCCTGCTCCTT	
F1 - 8004 - 8889	CCTCCCCCTGATTTGCCATT	60
R1	CTGTTAGGGGTCATGGGCTG	
F2 - 9600 - 10400	CTCCCCCTAATTACCCCCGA	59
R2	GGGGAGGCTGAGAAGTTTGT	
F3 - 10232 - 10623	GCCCTCTCCCTATGAACCCT	58
R 3	TAGGGGGAGAAGTCAGCAGG	

F4 - 14086 - 14602	CTGCCCGACTCCCTAATGTC	58
R4	AGTGGCAATGTCAGCAGTGT	
F5 - 14583 - 15218	ACACTGCTGACATTGCCACT	56
R5	TGAGGGGGTGTTACTAGGGG	
F 6- DLOOP1	TAACTCCCACCCCTAGCTCC	53
R 6- DLOOP1	GCATGGATCCCTAGCTCGAG	
F7 - DLOOP2	GAAACAGGAAAGCCTCGAGC	53,3
R7 - DLOOP2	TTGTGCTTACGGGGCTTTCT	
Combinação F10-R8004		54
Combinação F6-R7 (Old)		54
Combinação F16-R17		60

Suplemenatry table 2. Designed primers for Mitogenome amplification
